# Supplementary figures and images for: CG Methylation Covaries with Differential Gene Expression between Leaf and Floral Bud Tissues of Brachypodium distachyon
Source: PLoS One. 2016 Mar 7;11(3):e0150002. doi: 10.1371/journal.pone.0150002 (PMC4780816; doi:10.1371/journal.pone.0150002)

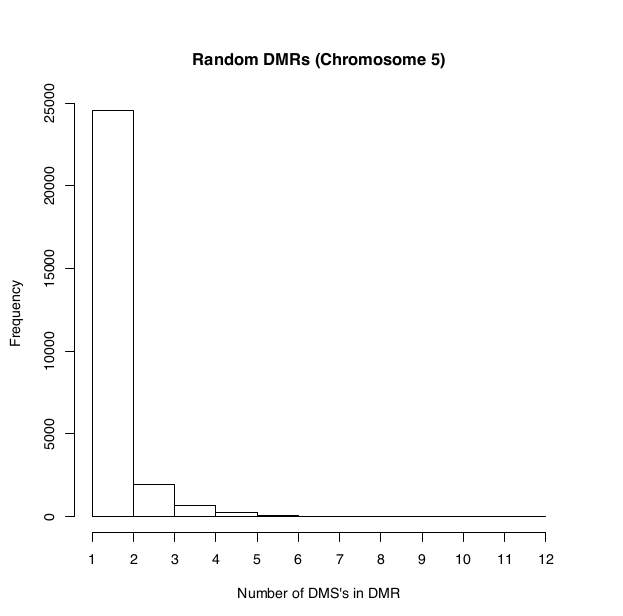

Supplement: S1 Fig — Methylated cytosines were randomized in the proper context, and the number of DMRs in the same direction were counted. Within a randomized genome, a run of five or more methylated cytosines in length represented 1.3% of all potential runs; we defined a DMR to be ≥ 5 methylated cytosines in the same direction, because this length represented a significant observation at the p ~0.01 threshold. See Materials and Methods for additional details. (TIF) [file pone.0150002.s001.tif]

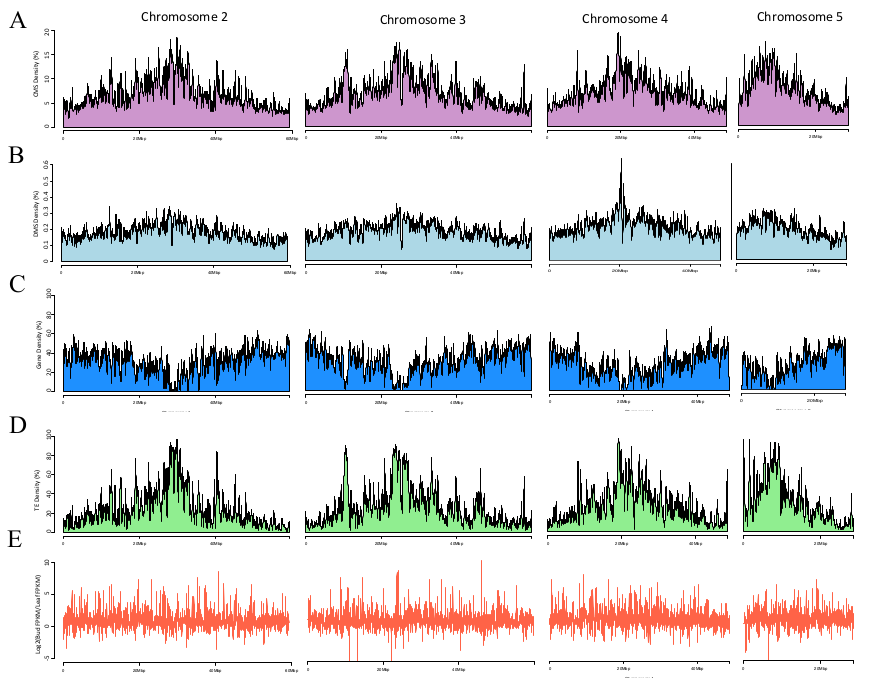

Supplement: S2 Fig — Plots of chromosomal densities of A) CMSs, B) DMSs, C) genes, and D) TEs. Density was measured within a 50kb sliding window for smoothing. E) The graphs plot differential gene expression plotted along the physical length of chromosomes. This figure mimics Fig 2 of the main text, but includes the remaining four chromosomes. (TIF) [file pone.0150002.s002.tif]

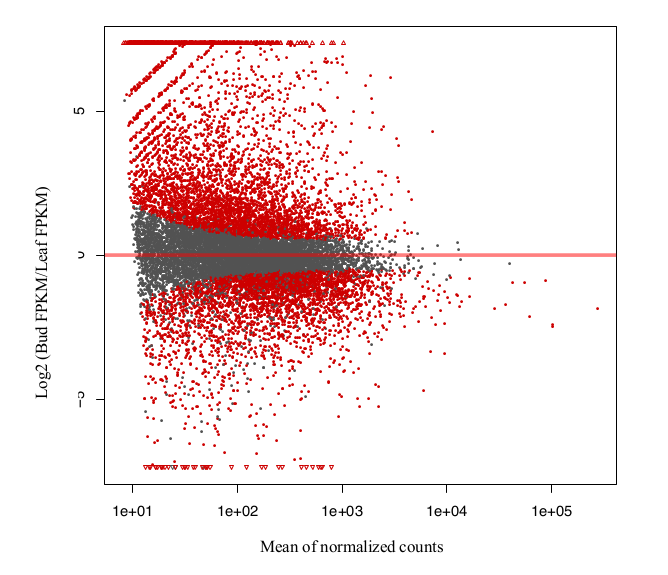

Supplement: S3 Fig — (TIF) [file pone.0150002.s003.tif]

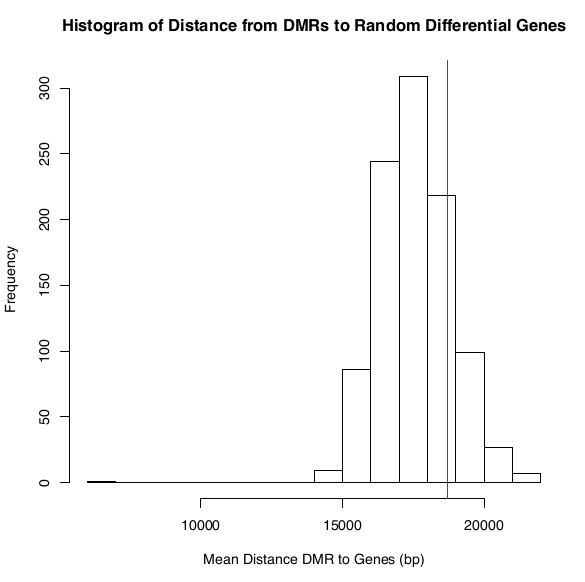

Supplement: S4 Fig — The histogram is based on 1000 randomizations (see Materials and Methods). The red line denotes the observed value. (TIF) [file pone.0150002.s004.tif]
