# Supplementary material for: CG Methylation Covaries with Differential Gene Expression between Leaf and Floral Bud Tissues of Brachypodium distachyon
Source: PLoS One. 2016 Mar 7;11(3):e0150002. doi: 10.1371/journal.pone.0150002 (PMC4780816; doi:10.1371/journal.pone.0150002)
Supplement: S1 Table — Estimates are based on analysis of chloroplast DNA in each replicate or on combined replicates. (DOCX) [file pone.0150002.s005.docx]

**S1 Table: The estimate of the rate of conversion error**. Estimates are based on analysis of chloroplast DNA in each replicate or on combined replicates.

| **Tissue/Replicate^1^** | **Error Rate** |
| --- | --- |
| Leaf 1 | 0.0133180 |
| Leaf 2 | 0.0106542 |
| Leaf 3 | 0.0111591 |
| Floral Bud 1 | 0.0086617 |
| Floral Bud 2 | 0.0098665 |
| Floral Bud 3 | 0.0094443 |
| L1+L2+L3 | 0.0115213 |
| F1+F2+F3 | 0.0092799 |

^1^ Estimates for individual replicates are from [35]. Estimates for combined replicates are from the sum of reads from all leaf and floral bud replicates.
