# Supplementary material for: CG Methylation Covaries with Differential Gene Expression between Leaf and Floral Bud Tissues of Brachypodium distachyon
Source: PLoS One. 2016 Mar 7;11(3):e0150002. doi: 10.1371/journal.pone.0150002 (PMC4780816; doi:10.1371/journal.pone.0150002)
Supplement: S2 Table — The table provides the number of reads after quality trimming, the number of reads that TopHat used to map for both left and right reads, and the maximum and minimum read lengths. The total number of transcripts is from based on output from cufflinks. (DOCX) [file pone.0150002.s006.docx]

**S2 Table: A summary of RNAseq data.** The table provides the number of reads after quality trimming, the number of reads that TopHat used to map for both left and right reads, and the maximum and minimum read lengths. The total number of transcripts is from based on output from cufflinks.

| **Replicate** | **No. In (Right)** | **No. Out (Right)** | **No. In (Left)** | **No. Out (Left)** | **Max Length** | **Min Length** | **Total** |
| --- | --- | --- | --- | --- | --- | --- | --- |
| Leaf 1 | 18,160,475 | 18,143,542 | 18,160,475 | 18,135,783 | 100 | 36 | 146,973 |
| Leaf 2 | 26,428,934 | 26,420,118 | 26,428,934 | 26,415,012 | 100 | 36 | 137,058 |
| Leaf 3 | 17,868,229 | 17,848,933 | 17,868,229 | 17,848,410 | 100 | 36 | 142,192 |
| Flower 1 | 14,734,746 | 14,723,047 | 14,734,746 | 14,720,678 | 100 | 36 | 163,786 |
| Flower 2 | 24,718,807 | 24,700,755 | 24,718,807 | 24,690,319 | 100 | 36 | 177,207 |
| Flower 3 | 12,599,231 | 12,580,503 | 12,599,231 | 12,578,360 | 100 | 36 | 160,370 |
