# Supplementary material for: CG Methylation Covaries with Differential Gene Expression between Leaf and Floral Bud Tissues of Brachypodium distachyon
Source: PLoS One. 2016 Mar 7;11(3):e0150002. doi: 10.1371/journal.pone.0150002 (PMC4780816; doi:10.1371/journal.pone.0150002)
Supplement: S3 Table — Only the enrichments terms with a p-value < 0.01 are given. (DOCX) [file pone.0150002.s007.docx]

**S3 Table: Go enrichment terms for differentially expressed genes between leaf and flower**. Only the enrichments terms with a p-value < 0.01 are given.

| **GO Term** |  | **P-value** |
| --- | --- | --- |
| GO:004867 | Serine-type endopeptidase inhibtor activity | 0.0054 |
| GO:0016706, GO:0010302 | Oxidoreductase activity, acting on paired donors, with incorporation or reduction of molecular oxygen, 2-oxoglutarate as one donor, and incorporation of one atom each of oxygen into both donors | 0.0010 |
| GO:0016307 | Phosphatidylinositol phosphate kinase activity | 0.0072 |
| GO:0046488, GO:0030384 | Phosphatidylinositol metabolic process | 0.0072 |
| GO:0005874 | Microtubule | 0.0028 |
| GO:0006184 | GTP catabolic process | 0.0006 |
| GO:0007017 | Microtubule-based process | 0.0020 |
| GO:0043234 | Protein complex C | 0.0007 |
| GO:0051258 | Protein polymerization | 0.0002 |
| GO:0044267 | Cellular protein metabolic process | 0.0087 |
| GO:0042626 | ATPase activity, coupled to transmembrane movement of substances | 0.0056 |
| GO:0006633, GO:0000037 | Fatty acid biosynthetic process | 0.0012 |
| GO:0007165, GO:0023033 | Signal transduction | 0.0029 |
| GO:0003777 | Microtubule motor activity | 1.418e-06 |
| GO:0007018 | Microtubule-based movement | 1.418e-06 |
| GO:0003924 | GTPase activity | 0.0086 |
| GO:0016887, GO:0004002 | ATPase activity | 0.0034 |
| GO:0004553, GO:0016800 | Hydrolase activity, hydrolyzing O-glycosyl compounds | 0.0027 |
| GO:0005975 | Carbohydrate metabolic process | 0.0002 |
| GO:0005840, GO:0033279 | Ribosome | 1.505e-14 |
| GO:0055085 | Transmembrane transport | 0.0055 |
| GO:0003735, GO:0003736, GO:0003738, GO:0003739, GO:0003740, GO:0003741, GO:0003742 | Structural constituents of ribosome | 8.320e-15 |
| GO:0006412, GO:0006416, GO:0006453, GO:0043037 | Translation | 1.987e-12 |
| GO:0016491 | Oxidoreductase activity | 0.0077 |
| GO:0016021 | Integral to membrane | 0.0096 |
| GO:0005622 | Intracellular | 2.428e-08 |
| GO:0004672, GO:0050222 | Protein kinase activity | 0.0003 |
| GO:0016020 | Membrane | 0.0012 |
| GO:0006468 | Protein phosphorylation | 0.0003 |
| GO:0055114 | Oxidation-reduction process | 0.0069 |
| GO:0005515, GO:0045308 | Protein binding | 0.0005 |
| GO:0005524 | ATP binding | 7.660e-11 |
