# Supplementary material for: CG Methylation Covaries with Differential Gene Expression between Leaf and Floral Bud Tissues of Brachypodium distachyon
Source: PLoS One. 2016 Mar 7;11(3):e0150002. doi: 10.1371/journal.pone.0150002 (PMC4780816; doi:10.1371/journal.pone.0150002)
Supplement: S4 Table — The models test for an effect of the TE distance to a gene and of TE methylation to differential gene expression. (DOCX) [file pone.0150002.s008.docx]

**S4 Table: Results of the application of linear models**. The models test for an effect of the TE distance to a gene and of TE methylation to differential gene expression.

|  | Coefficient Methylation | P-value^1^ | Coefficient Distance | P-value |
| --- | --- | --- | --- | --- |
| *Prop*C | -0.7233*** | **0.000118** | 3.895e-06 | 0.398970 |
| DMSs | -0.0193 | 0.102 | 4.040e-06 | 0.382 |

^1^ bolded values denote significance after sequential Bonferroni correction.
